# Exploring the Antimicrobial Activity of Sodium Titanate Nanotube Biomaterials in Combating Bone Infections: An In Vitro and In Vivo Study

**DOI:** 10.3390/antibiotics12050799

**Published:** 2023-04-22

**Authors:** Atiah H. Almalki, Walid Hamdy Hassan, Amany Belal, Ahmed Farghali, Romissaa M. Saleh, Abeer Enaiet Allah, Abdalla Abdelwahab, Sangmin Lee, Ahmed H.E. Hassan, Mohammed M. Ghoneim, Omeima Abdullah, Rehab Mahmoud, Fatma I. Abo El-Ela

**Affiliations:** 1 Department of Pharmaceutical Chemistry, College of Pharmacy, Taif University, Taif 21944, Saudi Arabia; 2 Bacteriology, Immunology and Mycology Department, Faculty of Veterinary Medicine, Beni-Suef University, Beni-Suef 62511, Egypt; 3 Materials Science and Nanotechnology Department, Faculty of Postgraduate Studies for Advanced Sciences, Beni-Suef University, Beni-Suef 62511, Egypt; 4 Department of Chemistry, Faculty of Science, Beni-Suef University, Beni-Suef 62511, Egypt; 5 Faculty of Science, Galala University, Sokhna, Suez 43511, Egypt; 6 Department of Fundamental Pharmaceutical Science, Graduate School, Kyung Hee University, 26 Kyungheedae-ro, Dongdaemun-gu, Seoul 02447, Republic of Korea; 7 Department of Regulatory Science, Graduated School, Kyung Hee University, 26 Kyungheedae-ro, Dongdaemun-gu, Seoul 02447, Republic of Korea; 8 Department of Medicinal Chemistry, Faculty of Pharmacy, Mansoura University, Mansoura 35516, Egypt; 9 Medicinal Chemistry Laboratory, College of Pharmacy, Kyung Hee University, 26 Kyungheedae-ro, Seoul 02447, Republic of Korea; 10 Department of Pharmacy Practice, College of Pharmacy, AlMaarefa University, Ad Diriyah, Riyadh 13713, Saudi Arabia; 11 Pharmacognosy and Medicinal Plants Department, Faculty of Pharmacy, Al-Azhar University, Cairo 11884, Egypt; 12 Pharmaceutical Chemistry Department, College of pharmacy, Umm Al-Qura University, Makkah 21955, Saudi Arabia; 13 Department of Pharmacology, Faculty of Veterinary Medicine, Beni-Suef University, Beni-Suef 62511, Egypt

**Keywords:** bone disorders, bone infections, sodium titanate nanotubes, anti-bacterial, anti-fungal, biomaterial, wound infections, rat model

## Abstract

The majority of bone and joint infections are caused by Gram-positive organisms, specifically staphylococci. Additionally, gram-negative organisms such as *E. coli* can infect various organs through infected wounds. Fungal arthritis is a rare condition, with examples including Mucormycosis (*Mucor rhizopus*). These infections are difficult to treat, making the use of novel antibacterial materials for bone diseases crucial. Sodium titanate nanotubes (NaTNTs) were synthesized using the hydrothermal method and characterized using a Field Emission Scanning Electron Microscope (FESEM), High-Resolution Transmission Electron Microscope (HRTEM), X-ray diffraction (XRD), Fourier-transform infrared spectroscopy (FTIR), Brunauer–Emmett–Teller (BET), and Zeta sizer. The antibacterial and antifungal activity of the NaTNT framework nanostructure was evaluated using Minimum Inhibitory Concentration (MIC), Minimum Bactericidal Concentration (MBC), Disc Diffusion assays for bacterial activity, and Minimum Fungicidal Concentration (MFC) for antifungal investigation. In addition to examining in vivo antibacterial activity in rats through wound induction and infection, pathogen counts and histological examinations were also conducted. In vitro and in vivo tests revealed that NaTNT has substantial antifungal and antibacterial effects on various bone-infected pathogens. In conclusion, current research indicates that NaTNT is an efficient antibacterial agent against a variety of microbial pathogenic bone diseases.

## 1. Introduction

The vast majority of bone and joint infections are caused by Gram-positive germs. In areas with limited blood supply, bone infections can be difficult to treat and often require prolonged antibiotic therapy in combination with surgical drainage or debridement. Ineffective or delayed therapy can result in severe morbidities such as pain, loss of function, and the need for additional surgery and antibiotics. Therefore, when selecting the most appropriate systemic antibiotic therapy, consideration must be given to factors such as the organism(s) isolated and their sensitivity profile, pharmacokinetic factors such as penetration into bone, the presence of prosthetic material, vascular supply of the affected limb, and the patient’s individual tolerance for the drugs [1].

*Staphylococcus aureus* is the most common bacterium responsible for osteomyelitis [2,3] and septic arthritis [4,5]. *Streptococcus pneumonia* and *Listeria monocytogenes* can also cause septic arthritis and raise concerns about underlying immune suppression [6,7,8]. Other microorganisms that may contribute to osteomyelitis in vasculopathic infections [9] include diabetic foot infections and septic arthritis caused by animal bites. Although certain patient populations are predisposed to Gram-negative infections, they account for a small proportion of bone and joint infections. Prior to the introduction of the Hib vaccine, *Haemophilus influenza* was a leading cause of septic arthritic joints in preschool children, but this is now less common [10].

Fungal infections are a rare but significant cause of osteomyelitis and arthritis. The most common fungal diseases that cause osteomyelitis are Candida, Aspergillosis, and Mucormycosis. Osteomyelitis and arthritis induced by mucormycetes are uncommon conditions that are among the most difficult consequences in orthopedic and trauma surgery [11]. The epidemiology of these musculoskeletal manifestations of fungal infections is a significant problem in both animals and humans. The Candida species are widespread yeasts. *Candida albicans* is a common human commensal, and other species can survive in non-living conditions such as soil. Aspergillus species infections of the bone and joints are characterized by a poor clinical prognosis and complex neurological consequences. Host-predisposing factors include immunosuppression, intravenous drug use, chronic underlying illnesses, and past surgical operations. Nosocomial diseases can be transmitted by polluted air ventilation systems or water pipes. *Aspergillus fumigatus* is the most common pathogen, followed by *Aspergillus flavus* and A. niger, but focused and customized antifungal medication is necessary [12]. Since the development of antibiotic therapy in the 1940s, particularly with the widespread use of immunosuppression and parenteral lines, candidiasis and mucormycosis have contributed to an increase in mucocutaneous and deep-organ infections [13].

Sodium titanate is an inorganic ion exchanger with a strong affinity for numerous metals. It acts effectively in mediums with different pH levels, either highly alkaline, neutral, or mildly acidic fluids. Titanate has been utilized in numerous applications [14]. Their electrophoretic deposition allows them to be supported on solid substrates [15]. Zhou and Yu developed super hydrophilic surfaces through the electrophoretic deposition of titanate [16]. They are also effective against Gram-positive and Gram-negative pathogens, such as *Staphylococcus aureus* and *Escherichia coli* [14]. These bacteria are among the most prevalent causes of nosocomial infections and are transmissible through contaminated surfaces [14]. Additionally, bacterial adherence to surfaces is an essential step in the creation of hazardous biofilms [17]. Inhibiting early bacterial adherence is therefore a promising antibacterial strategy [18]. 

Humans are often infected by microbes such as bacteria, fungi, and viruses throughout everyday life. The use of antimicrobial agents is crucial for imparting sterility (e.g., hospital trays) and preventing infection (e.g., wound dressing) one route from a bone infection. Antibacterial compounds incorporating diverse natural and inorganic substrates have been thoroughly studied [19]. In comparison to other antimicrobial compounds, TiO_2_ has garnered greater attention owing to its favorable qualities, such as excellent stability, environmental friendliness, safety, and broad-spectrum antibiosis [20]. Using fine TiO_2_ particles in antibacterial formulations has been the subject of a great deal of research. Yet, the powder TiO_2_ catalyst has the disadvantage of slurry post-application separation [20]. Therefore, surface-immobilized TiO_2_ with a large specific surface area is more promising for antibacterial applications [21], which is why we prepared it in nanotube formulations. Therefore, in this study, the modification of titanium in the form of Na TNT is an in vitro and in vivo study on both bacterial and fungal infections that need to be investigated.

The purpose of this work was to examine the antibacterial and antifungal activities of a sodium titanate biomaterial generated by the hydrothermal process against a variety of infections that affect bone in humans and animals.

## 2. Materials and Methods

### 2.1. Materials

Strains: All fungal isolates used in this investigation were obtained from the reference collection of the Fungal Research Institute (Doki, Giza, Cairo, Egypt), while the bacterial strains were obtained from the American Type Culture Collection (ATCC) at the Cairo Microbiology Research Center. Strains used for phylogenetic reconstruction represented all relevant Mucorales taxa, including the clinically significant species, CNRMA 03894 *Mucor rhizopus*, which was used in this study. This study examined the effects of NaTNT as a preventive agent against wound infection for osteomyelitis and bone infection prevention. The reference standard for doxorubicin was supplied by Pharma Swede Pharmaceutical Company, while cyclohexamide served as the standard antifungal medication. Gram-positive strains including *St. pneumonia* (ATCC 49619), *S. aureus* (ATCC 25913), *Listeria monocytogenes* (ATCC 19115), and Gram-negative strains including *E. coli* (ATCC 25922), *Haemophilus influenza* (ATCC 49766), and *Bacillus subtilis* were utilized for antibacterial research (ATCC 35021). Regional Center for Mycology and Biotechnology (RCMB) isolates for Aspergillosis included *Aspergillus flavus* RCMB 02783, *Aspergillus fumigatus* RCMB 02564, and *Aspergillus niger* RCMB 02588, *Candida albicans* RCMB 05035 for Candidacies, *Mucor rhizopus* CNRMA 03.894 for Mucormycosis, and *Pencillieum notatum* (NCPF 2881). The bacterial isolates were incubated at 37 °C for 24 h using Muller Hinton broth and Muller Hinton Agar for bacterial growth. For fungal growth, Sabaroud Dextrose Agar and broth were employed with the conventional antifungal medication (cyclohexamide) at 25 °C for five days. Before the experiments, each tube was sanitized using an autoclave. 

### 2.2. Synthesis of Sodium Titanate Nanotubes

All chemicals involved in the preparation step were of analytical grade and obtained from Sigma Aldrich Co. They were used without further purification. Na-TNT was synthesized using the hydrothermal method [22]. Firstly, 10 g of anatase TiO_2_ powder was stirred for 30 min with 500 mL of 10.0 M NaOH until a milky suspension appeared. This milky suspension was allowed to react hydrothermally in a Teflon-lined stainless-steel autoclave with a 500 mL volume for 23 h at 160 °C to produce Na-TNT. The autoclave was then cooled to ambient temperature. Afterward, the prepared Na-TNT was filtered, washed several times with distilled water, and finally dried at 80 °C overnight.

Characterization of Na-TNT was performed using several techniques. PANalytical (Empyrean) XRD using Cu Ka radiation (wavelength = 0.154 cm^−1^) was used to obtain the X-ray diffraction pattern at an accelerating voltage of 40 kV, a current of 35 mA, scan angle ranging from 5 to 80°, and scan step of 0.04°. The FT-IR spectrum was measured in the range of 400–4000 cm^−1^ on a VERTEX 70 FT-IR spectrometer (Bruker Optics, Ettlingen, Germany) via the KBr pellet technique. Zeta potential was detected using Zetasizer Nano-ZS90 (Malvern, UK). Quanta FEG 250 (Thermo Fisher Scientific, Basel, Switzerland) electron microscope was used for FESEM imaging. High-resolution transmission electron microscope (HRTEM) imaging was performed using a JEOL-JEM 2100 (Tokyo, Japan) electron microscope operating at 200 kV. Brunauer-Emmett-Teller (BET) surface area was detected by the N2 sorption technique using Micromeritics TriStar II.

### 2.3. Anti-Microbial Measurements

#### 2.3.1. Fungal Isolates and Bacterial Inoculum Preparations

Recent cultures of *Aspergillus flavus* RCMB 02782, *Aspergillus fumigatus* RCMB 02564, *Aspergillus niger* RCMB 02568, *Candida albicans* RCMB 05035, *Mucor rhizopus* CNRMA 03.894, and *Pencillieum notatum* (NCPF 2881) were used to create suspensions, which were plated on Sabouraud’s Dextrose Agar (SDA). After incubation, about 4–5 yeast colonies were transferred (using a sterile loop) to test tubes containing 5 mL of 0.9% saline solution. The final inoculum’s turbidity was standardized using a suspension of barium sulphate 1.175% and sulphuric acid 1% (tube 0.5 on the McFarland scale, turbidity or standard tube). The final concentration was approximately 1.5 × 10^8^ colony-forming units per milliliter (CFU/mL) [23]. 

To prepare the bacterial inoculum, *St. pneumonia* (ATCC 49619), *S. aureus* (ATCC 25913), *Listeria monocytogenes* (ATCC 19115), Gram-negative *E. coli* (ATCC 25922), *Haemophilus influenza* (ATCC 49766), and *Bacillus subtilis* (ATCC 35021) cultures were plated on Muller Hinton agar media and incubated at 37 °C for 24 h. About six colonies were then transferred to saline to obtain 10^8^ CFU/mL, using conventional tube matching, as described in fungal inoculum preparation.

To determine antibacterial activity, the microdilution susceptibility test in Muller–Hinton Broth (Oxoid) was employed to estimate the minimal inhibitory concentration (MIC) for bacteria. Sabouraud’s Liquid Medium (Oxoid) was used to estimate the MIC for fungus. Stock-tested chemical solutions were prepared in saline. The stock solution was subsequently diluted with standard method broth (Difco) to manufacture twofold serial dilutions of the broth containing approximately 10^8^ CFU/mL of test microorganisms. The solution was then applied to each well of the 96-well Microtiter plate. The microplates were sealed and incubated for 24 h at 37 °C in a humid room. Prior to the conclusion of the incubation period, the MIC values were determined to be the lowest concentrations of the chemical that did not produce visible turbidity. Uninoculated medium served as the control in studies conducted under the same conditions as the test chemicals. To confirm their reproducibility, each experiment was conducted three times. Means and standard deviation (SD) values were calculated using SPSS version 21, and *p*-values less than 0.05 were considered statistically significant.

#### 2.3.2. Minimal Bactericidal Concentration (MBC)

On Muller Hinton Agar Plates, the MIC dilution and at least two of the more concentrated tested NaTNT dilutions are plated and viable CFU/mL are determined. MBC is the lowest concentration of NaTNT at which no viable bacterial colonies are seen following a 24 h incubation at 37 °C (bactericidal activity).

#### 2.3.3. Minimum Inhibitory Concentration for Fungal Isolates (MIC-_f_)

Using the broth microdilution method to determine the MIC of fungi [23]. 100 µL of Sabouraud’s Dextrose broth medium (SDB) was dispensed into each well of a 96-well microdilution plate with a “U” shaped bottom. Then, 100 µl of the tested nanomaterials emulsion was added to the first horizontal row of plate wells. A 100 µL aliquot extracted from the most concentrated well and transferred to the next well resulted in concentrations ranging from 1000 to 1.9 µg/mL. In the end, 10 µL of an inoculum suspension containing different tested strains was put into each well of the plate, where each column represented a fungal strain. In the presence of the conventional antifungal Cyclohexamide standard antifungal treatment for rapid-growing fungi were a positive control (medium without fungal strains) and a negative control (fungal strains without Na TNT). On SDB plates, each well contained 100 µL of SDB with various concentrations of tested nanomaterials in 2-fold serial dilutions (1000, 500, 250, 125, 62.5, 31.25, 15.62, 7.81, 3.95, and 1.95 µg/mL). A total of 10 µL solutions containing 1.5 × 10^8^ fungal strains/mL were injected after dilution.

The U-shaped plates were incubated at 25 °C for 72 h. After the required period of incubation, the presence (or lack) of growth was visually evaluated. Consideration was given to the creation of cell clusters or “buttons” in the plate wells. The MIC was determined as the lowest concentration that inhibited fungal growth visibly. According to the criteria established by Morales et al., the antibacterial activity of the nanomaterials tested was evaluated (considered active or inactive) [24]: Strong/excellent activity (MIC < 1000 µg/mL).

### 2.4. Sorbitol Assay-Effect of NaTNT on the Cell Wall of Different Tested Fungal Strains

The assay was conducted using sorbitol-containing and sorbitol-free (control) media to examine potential antifungal processes involved in the nanomaterials’ effect on the cell walls of various fungi. The culture medium (peptone water medium) was supplemented with sorbitol at a concentration of 0.8 M sorbitol (5 g/L added to peptone water media 15 g/L). The test was conducted using the microdilution technique in “U”-shaped 96-well plates. The plates were aseptically sealed and incubated at 35 °C, and readings were obtained on the fifth day of incubation. Based on the ability of sorbitol to act as an osmotic protective agent for the fungal cell wall, the higher MIC values reported in the medium with added sorbitol compared to the standard medium suggested that the cell wall is one of the potential cell targets for the NaTNT. Cyclohexamide was utilized as the placebo. The assay was run in triplicate and the findings were represented as the arithmetic mean [25].

#### 2.4.1. Minimum Fungicidal Concentration Assay (MFC)

To evaluate the MFC, we inoculated SDA-coated Petri dishes with 100 µL aliquots of MIC, MIC 2, and MIC 4 of the investigated nanomaterials, cyclohexamide, and the negative control for fungal growth. After 72 h of incubation at 25 °C, the MFC was evaluated based on the development of the control organisms. The minimal fungicidal concentration (MFC) was defined as the lowest product concentration that prevented the growth of the tested microorganisms, resulting in either 50 or 99.9% fungicidal activity [26]. Assays of biological activity were conducted in triplicate, and the results were represented as the arithmetic mean of the MIC and MFC concentrations. Using both dilution procedures, it was feasible to identify whether the chemical is active; however, it is not possible to determine whether the substance will kill the fungus or simply slow its growth. The minimum fungicidal concentration (MFC) test is performed for this purpose. Small aliquots from each broth dilution test are subcultured on a rich solid medium and incubated for a predetermined amount of time and temperature, depending on the tested fungus species. According to papers standardized by the Chemical and Laboratory Standard Institute (CLSI), MFC is regarded as the lowest concentration of the drug in which no observed subculture growth occurs. MFC could also provide information regarding fungicide or fungiostatic activity. If the MFC and MIC are the same, the substance is a compound fungicide; if the MFC is higher than the MIC, it is fungiostatic [27]. 

#### 2.4.2. Disc Diffusion Assay

For the standard size (50 mm diameter) disc diffusion analysis of all tested microorganisms, Whatman filter paper discs were made and then stored in 10 screw-capped wide-mouthed containers to assure sterilization. The bottles were then placed in a 150 °C hot air oven. Following this, the standard discs of the sterilized filter paper were impregnated with a 1000 µg/mL solution of the test substance (NaTNT) in saline. Then, they were placed in duplicate on nutrient agar plates seeded with the appropriate test organism. For the antibacterial assay, the usual conditions of 10^8^ CFU/mL (Colony Forming Units per milliliter) were applied. Utilizing Petri dishes with a diameter of 12 cm, two discs of filter paper were inoculated in each dish. Gram-positive *St. pneumonia* (ATCC 49619), *S. aureus* (ATCC 25913), *Listeria monocytogenes* (ATCC 19115), and Gram-negative *E. coli* (ATCC 25922), *Haemophillus influenza* (ATCC 49766), and *Bacillus subtilis* were utilized as test organisms (ATCC 35021). Doxycycline was used as a reference antibiotic against Gram-negative bacteria, Gram-positive bacteria, and fungi, in that order. For bacteria and fungi, the plates were incubated at 37 °C for 24 h and at 25 °C for 5 days, respectively. The twofold serial dilution approach revealed a considerable growth inhibition zone for the derivative.

#### 2.4.3. Agar Diffusion Method for Fungal Isolates

The Agar diffusion technique is a semi-quantitative assay that involves applying a sample with a known concentration to a Sabaroud Agar Surface that has been inoculated with a standard number of fungal cells (*Aspergillus flavus* RCMB 02782, *Aspergillus fumigatus* RCMB 02564, and *Aspergillus niger* RCMB 02782), *Candidacies* (*Candida albicans* RCMB 05). Various procedures can be used to apply the sample, including disc diffusion, in which discs made of sterile filter paper (6 mm) are soaked with the sample and then applied to the agar surface in the presence of cyclohexamide, the standard antifungal. Following inoculation, the samples disperse into the agar medium, forming a circular concentration gradient. If the sample exhibits antifungal action, a zone of growth inhibition will emerge around the disc as the fungus multiplies. This inhibitory zone is measured in millimeters, and it is categorized by some writers as entire inhibition, partial inhibition, or no inhibition [28,29].

#### 2.4.4. Antifungal Assay

Agar dilution method for detecting antifungal activity of various tested nanomaterials. According to the method of eff-Agboola et al. [30], the antifungal efficacy of NaTNT against randomly selected fungus isolates was examined after 72 h of growth on SDA at 25 degrees Celsius, the examined fungi were suspended in physiological saline (0.9% NaCl), and adjusted to 1.5 × 10^8^ CFU. After preparing and autoclaving SDA at 121 °C for 15 min and storing it at 55 °C, the tested nanomaterials were created and mixed with SDA based on the concentration tested. NaTNT was prepared at 1, 2, and 3% concentrations. The 20 mL of solidified Sabaroud-agar medium was then put into sterilized Petri dishes. On the agar plates, equal volumes of the fungal suspensions were inoculated and speared. The plates were then incubated for 72 h at 25 °C before being evaluated on the fifth day of incubation. 

### 2.5. In Vivo Study with Wound Healing, Antimicrobial Evaluation, and Histopathological Investigations

Antibacterial efficacy is determined by the best in vitro *(S. aureus* and *M. indices)* and in vivo (wound healing) results from this study (*S. aureus* and *M. indices*). The Institutional Animal Care and Use Committee (IACUC) of Beni-Suef University approved all animal experiments for research and testing. All animal handling, care, infliction of wounds, and treatment were conducted in accordance with the IACUC’s requirements for approval. A total of 24 six-week-old adult male rats weighing between 150 and 200 gm were purchased from the faculty of pharmacy lab animals at Beni-Suef University. Each group consisted of three rats and was divided into eight groups (4 groups for *S. aureus* and 4 groups for *M. indices*). The rats were separated into four different groups as follows; Group 1 (G1) were control negative non-infected normal rats, group 2 (G2) were treated with Na TNT ointment with 10% Vaseline, group 3 (G3) rats were treated with commercial standard ointment as woundplast (fucidin) for *S. aureus* infection or with cyclohexamide ointment for *M. Rhizopus* infection, and finally G4 were control positive infected non-treated rats in both tested microorganisms. After anaesthesia with ketamine (90 mg/kg b.wt.) and xylazine (5 mg/kg b.wt.), we used a 1:1 mL ratio and injected 0.1 mg/100 gm b.wt., intraperiotineally; subsequently, after induction of an 1 × 1 cm wound area (at the back in *S. aureus* and in the top leg surface in *M. indices)*, 100 µL of *S. aureus* and *M. indices* (1 × 10^8^ CFU/mL) were administered to the wounds. At the wound site of the treated groups, the final concentration of NaTNT ointment was 10 gm:90 gm Vaseline. Rates of wound contraction were measured daily and every three days until the 12th day. All animals are maintained in sanitary and regulated circumstances. All rats were examined on a daily basis for wound fluid, signs of infection, and other abnormalities. In order to evaluate wound-healing activity, wound contraction % and wound closure time were utilized. On days Zero, 3, 6, 9, and 12 following surgery, the size of the wound was measured [31] (Equation (1)).

(1) $$\;\;\;\;\;\;\mathrm{Percentage}\;\mathrm{of}\;\mathrm{woundsize}\;=\frac{\mathrm{Wound}\;\mathrm{area}\;\mathrm{on}\;\mathrm{day}\;X}{\;\mathrm{Wound}\;\mathrm{area}\;\mathrm{on}\;\mathrm{day}\;\mathrm{zero}}\times 100$$

At 2, 6, and 12 days of therapy, samples of infected and treated wounds were collected in Muller Hinton broth media and incubated at 37 °C for 24 h. Then, the spread-plate method was used to determine the quantity of bacteria in solution, whereas for fungi, the plate count method was applied (*Mucor rhizopus*). The shape of the fungal isolate cultivated from debrided tissue was evaluated on potato dextrose agar (PDA) at 30 °C in the dark, and temperature tolerance was evaluated on Sabouraud’s dextrose agar (SAB). There was no growth at 40 degrees Celsius, which was the optimal temperature. After 72 h at 30 °C, the diameter of the colonies was roughly 6.3 cm. The colonies on PDA were a light brown color with elevated mycelia. A microscopic inspection revealed yellowish-brown sporangia with walls that were finely roughened, ranging in diameter from 35 to 75 μm. The rats were photographed at 3, 6, and 12 days and slaughtered so that the wound tissues could be extracted for H&E staining and histological examinations using 10% formalin. The processed samples and acquired sections (5 mm) were stained with hematoxylin and eosin (H&E) and analyzed microscopically for cellular or immunological infiltration or macrophages in the wound region, collagen or fibroblast percentage, and vascularization rate [32].

### 2.6. Statistical Analysis

The data were presented as the mean standard deviation of the mean (S.E.M.). According to Snedecor, statistical significance was assessed using a one-way analysis of variance (ANOVA) [33]. Tukey’s post-hoc test for multiple comparisons was then performed using SPSS (version 20.0) software (IBM SPSS Statistic 20.0, Armonk, NY, USA). *p*-values below 0.05 were deemed statistically significant.

## 3. Results and Discussion

### 3.1. Nano Material Characterization 

**Figure 1:**
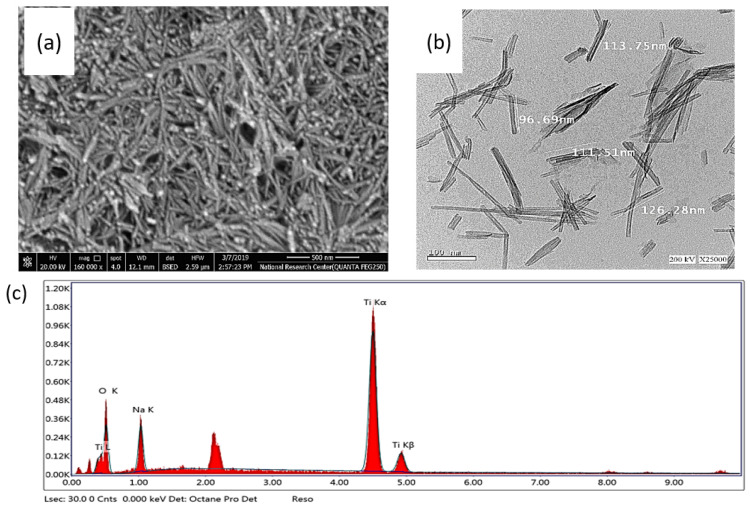
FESEM (**a**), HRTEM (**b**) and EDX elemental composition of Na-TNT (**c**).

Figure 1 presents the HRTEM and FESEM images of Na-TNT. The images reveal that the synthesized nanotubes are partially agglomerated to a low extent and are relatively thick and smooth. The HRTEM images show multiwall open-ended nanotubes with an average length between 100 and 200 nm. The inner diameter of the prepared nanotubes is about 4 nm with an outer diameter of about 10 nm. These findings support that alkaline hydrothermal treatment is an efficient method for converting nano-particulate morphology into a nanotubular structure. Additionally, EDX analysis confirmed the presence of Ti, O, and Na elements in the prepared nanotubes, as shown in Figure 1.

**Figure 2:**
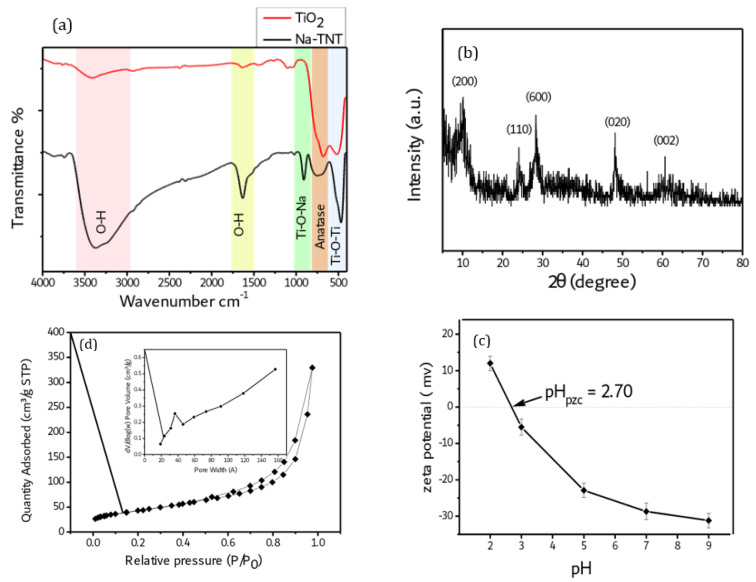
FT-IR Spectra (**a**), XRD pattern (**b**), nitrogen adsorption/desorption isotherm with inset pore size distribution curve (**c**) and Zeta potential (**d**) all for Na-TNT.

Figure 2a represents the FT-IR spectra of TiO_2_ and Na-TNT. The FT-IR spectrum of Na-TNT shows the same main peaks of TiO_2_ with increased intensity for peaks corresponding to the hydroxyl groups of Na-TNT. The broad peak appears at around 3370.0 cm^−1^ and corresponds to the O-H stretching vibration, indicating the presence of a high number of O-H functional groups in Na-TNT. The strong adsorption band at 1630 cm^−1^ is attributed to the bending vibration of hydroxyl groups [34]. Another characteristic peak that appears at 910 cm^−1^ is assigned to the Ti-O-Na bond [35]. The peak appears around the wave number of 730 cm^−1^ and reflects the anatase phase of titanate. Furthermore, the peak around 466 cm^−1^ could be attributed to the Ti-O-Ti crystal phonons due to the tubular structure of Na-TNT [36]. 

Figure 2b illustrates the XRD pattern of Na-TNT, which shows peaks centered at 2θ values of 9.9°, 24.4°, 28.4°, 48.2°, and 61.9°, corresponding to the crystal planes (200), (110), (600), (020), and (002), respectively. These results confirm the formation of Na-TNT with an orthorhombic unit cell, according to ICDD card no 00-057-0123 [22]. The prepared sample shows a crystallite size of 14.7 nm, according to the XRD results.

Further investigation of Na-TNT was performed using N2 sorption analysis to identify the porous nature of the material and its specific surface area. Figure 2c presents the nitrogen adsorption/desorption isotherms of Na-TNT with a pore size distribution curve as an inset figure. The adsorption isotherm of Na-TNT shows a type IV isotherm pattern with a noticeable hysteresis loop, reflecting the mesoporous nature of Na-TNT. According to the IUPAC classification, the hysteresis loop of Na-TNT can be classified as a combination of H1 and H3 types. The H1 type hysteresis loop is characteristic of mesoporous materials that have a narrow range of uniform cylindrical-like pores, reflecting the tubular structure of Na-TNT. On the other hand, the H3 type refers to a wide range of slit-shaped pores with non-uniform size; such pores arise from solids consisting of non-rigid agglomerates or aggregates of plate-like particles [37]. The pore size distribution curve shows two types of pores: the first type is a narrow range of pores around 4 nm, which refers to the pores inside the nanotubes and is in good agreement with the inner diameter obtained from the HRTEM images, while the second type is a broad distribution of larger pores that may be attributed to the pores between the nanotubes. The shape of the isotherm of Na-TNT, as well as its hysteresis loop, are in agreement with those previously published for titanate nanotubes [38]. Furthermore, the textural properties are summarized in Table 1.

According to the zeta potential results of several previous studies, the TiO_2_ surface is positively charged in the acidic medium and negatively charged in and near an alkaline medium with an isoelectric pH (pH_PZC_). The zeta potential of Na-TNT as a function of pH was measured at room temperature and the results are indicated in Figure 2d. The results indicate that the synthesized material is positively charged in the low pH medium (pH = 2) [39]. At a low pH solution (pH = 2), a high concentration of H^+^ leads to a protonation reaction of hydroxyl groups on the surface of the material, giving rise to positively charged particles at this pH, while at pH 3–9, Na-TNT is negatively charged as a result of a deprotonation reaction at this pH. Chemical reactions that occur on the surface of the particles are described in Equations (2) and (3).

The isoelectric pH (pH_PZC_) value of Na-TNT is 2.7, implying particles of a neutral surface at this pH. Overall, the zeta potential results revealed the formation of a dominant-negative charge on the surface of Na-TNT particles at the pH range from 3 to 9.

(2) $$–\mathrm{Ti}–\mathrm{OH}+H^{+}\to \;–Ti–OH_{2}^{+}\;\;(\mathrm{protonation}\;\mathrm{reaction})\;$$

(3) $$–\mathrm{Ti}–\mathrm{OH}+{\mathrm{OH}}^{-}\to \;–\mathrm{Ti}–O^{-}+H_{2}O\;\;(\mathrm{deprotonation}\;\mathrm{reaction})\;$$

### 3.2. Antimicrobial Investigations

#### Anti-Bacterial Assay

In terms of comparative efficacy and bone penetration, investigations on animals have shed light on the management of bone infections. The absence of debridement in the animals, the large initial inoculum, and the lack of experience with recurring or persistent infection are limitations of these studies. Similarly, experimental models do not permit long-term follow-up. Invariably, *S. aureus* is chosen as the infecting organism to research anti-Gram-positive drugs, which is representative of many but not all patient infections; hence, the results cannot be immediately applicable to streptococcal infections. The relevance of peak bone concentrations in relation to the MIC for the isolate is not well understood, and this does not necessarily match with the clinical outcome in animal investigations. In spite of these restrictions, useful data about antibiotic bone and serum concentrations, time to sterilization, and percentage cure have been published. There will be a discussion of studies on the most frequently used antibiotics for bone and joint infections.

**Figure 3:**
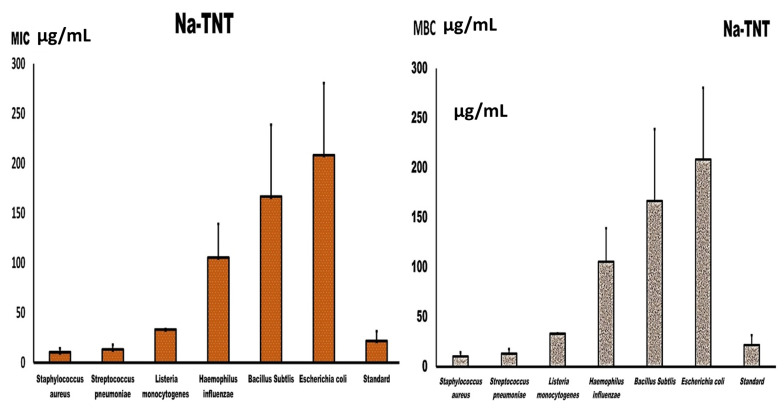
Presents the MIC and MBC values of NaTNT against both Gram-positive bacteria and Gram-negative bacteria (Mean ± SE). The best obtained results as shown by the MIC and MBC was achieved by Na TNT against *S. aureus*, *St. pyogens*, and *L. monocytogenes*.

The MIC and MBC of the examined NaTNT against different bacterial isolates are depicted in Figure 3. The figure reveals that the MIC value of the tested NaTNT varied significantly from one species to the next. Additionally, the recorded values of MBC and MIC are comparable for *L. monocytogenes*, indicating its bactericidal activity; however, they were significantly different for *S. aureus, St. Pyogens*, and the tested Gram-negative species. The maximum MIC value was slightly above 250 µg/mL and was recorded for *E. coli*, followed by *B. subtilis* and *H. influenza*, while the minimum MIC value was approximately 15 µg/mL for *Staphylococcus aureus* and *St. pneumoniae*. Therefore, *Staphylococcus aureus*, *Streptococcus pneumoniae*, and *Listeria monocytogenes* had better values for MIC than *E. coli*, *B. subtilis*, and *H. influenza*. Similarly, for MBC, *E. coli* also had the highest MBC value, followed by *B. subtilis* and *H. influenza*, while *Staphylococcus aureus* and *Streptococcus pneumoniae* had the lowest MBC values followed by *L. monocytogenes*, which shows the bactericidal action of NaTNT, as shown in Figure 3.

**Figure 4:**
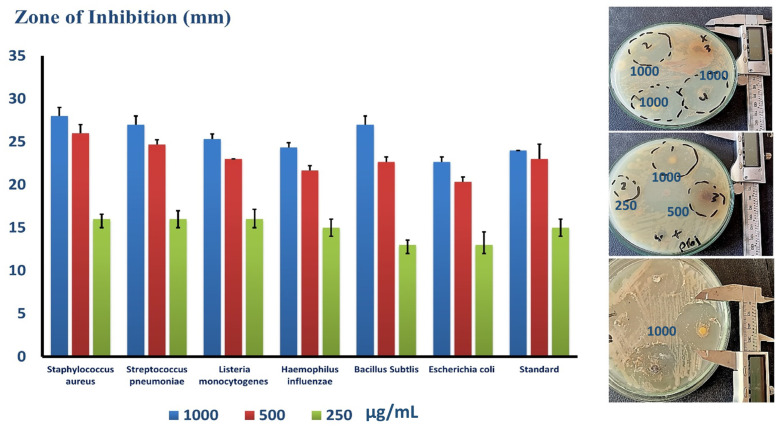
Illustrates the calculated mean of the inhibition zone (mm) at different concentrations of NaTNT versus diverse species of bacteria, and the mean of the inhibition zone against standard antibiotics (Doxycycline for both Gram-negative and Gram-positive) (Mean ± SE). Representative clear zone of inhibition appeared in the agar plates. “1–4”: the tested different concentrations used for NaTNT as a key on the plates which meet (1000, 500 & 250 µg/mL).

Plates containing several strains of bacteria, including Gram-positive *St. pneumonia* (ATCC 49619), *S. aureus* (ATCC 25913), *Listeria monocytogenes* (ATCC 19115), and Gram-negative *E. coli* (ATCC 25922), *Haemophillus influenza* (ATCC 49766), and Bacillus subtilis (ATCC 35021). In addition, the figure illustrates the inhibition zone of each strain at different NaTNT doses. The inhibition zone was determined by the Agar diffusion technique in millimeters. Overall, the measured diameters of different species were distinct. As such, Figure 4 is a bar graph depicting the computed mean of the inhibition zone (mm) at various concentrations of NaTNT (1000, 500, 250, and 125 µg/mL) vs. the distinct types of bacteria stated before on the X-axis. Overall, the effect of NaTNT on the examined strains was inconsistent. Regarding Gram-positive bacteria, *S. aureus*, *Bacillus subtilis*, and Staphylococcus had the highest response rates, while *L. monocytogenes* had the lowest. Second, the reaction of Gram-negative H. influenza was greater than that of *E. coil*. In addition, the picture illustrates that the inhibition zone was precisely proportional to the NaTNT concentration in all tested species. *S. aureus* had the largest inhibitory zone, measuring roughly 28.5 mm, while *L. monocytogenes* exhibited the smallest, measuring around 26 mm. Notably, the tested NaTNT was compared to doxycycline for both Gram-negative and Gram-positive organisms (24 mm), and the results revealed that the effect of NaTNT and the compared standard drug was hardly the same in both Gram-negative and positive organisms, with NaTNT being more effective against *Bacillus subtilis*. From this perspective, the researched NaTNT could serve as an effective alternative to traditional antibiotics in combating bacterial resistance.

Regarding the fungal broth micro dilution assay for NaTNT, a number of studies have explored the interaction of nanomaterials with bacteria, but few have examined their impact on fungi. This may be related to the comparative simplicity of bacterial and fungal systems [40].

The Mucor strain is one of the most severely affected fungi by NaTNT, according to this study’s antifungal examination of NaTNT. Comparable to other invasive fungal illnesses, Mucor contagious species cause immunosuppression (especially delayed and excessive neutropenia, a serious hematological disease with or without stem cell transplantation, and the delayed use of corticosteroids) that predisposes to Mucormycosis [41]. Inadequately treated diabetes mellitus with or without diabetic ketoacidosis, iron overload, and medication with the iron chelator deferoxamine are additional risk factors [42]. Mucormycosis’ most prevalent mode of transmission is the inhalation of fungal spores, manifesting as aspiratory or rhino-orbito-cerebral forms. Diseases caused by Mucorales spp. have been monitored for expansion following significant injuries, including burns [43], induced by direct spore inoculation into the tissue. In certain instances, infections can arise without other risk factors. In rare instances, spores infiltrated the tissue through tiny injuries like insect bites or animal scratches [44]. Mucormycosis of the gastrointestinal tract has also been described, particularly from swallowed spores [45]. A significant characteristic shared by all varieties of mucormycosis is the invasion of blood vessels and consequent thrombosis and necrosis of tissue. Angio-invasion also explains the often seen spread of infection in Mucormycosis [46]. Mucormycosis is distinguished from other forms of illnesses, such as conspicuous aspergillosis, by its unique histological alterations, its rapidly dynamic character, and the widespread tissue rot that often accompanies it. In addition, diabetes, press overload, and deferoxamine medication are unique risk factors for Mucormycosis. Due to the threat posed by this fungus, it is essential to seek innovative antifungal substances that may cure or prevent this sort of severe fungal infection.

**Figure 5:**
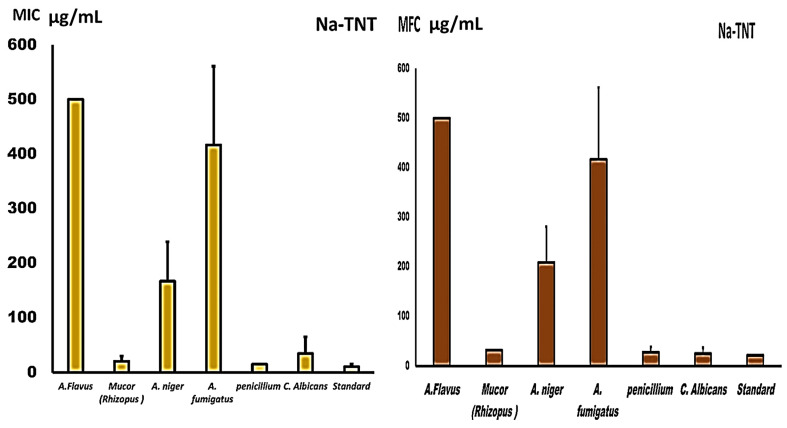
Presents the MIC and MFC values (µg/mL) of NaTNT against both different fungal isolates (Mean ± SE). The best obtained results for NA TNT were obtained against Mucor and pencillieum species while fumigatus and flavus needed higher concentrations.

The MICs for *A. niger* and *Fumigates*, other than *C. albicans*, were identical to the MFCs, confirming the fungicidal action of NaTNT, as shown in Figure 5, but were somewhat higher for the other examined isolates. The nanomaterial NaTNT proved efficient against all fungal species, particularly Mucor and Penicillium strains (20 and 15 µg/mL, respectively). In higher doses, they exhibited antifungal action against additional species, such as Candida (66 µg/mL) and *A. niger* (125 µg/mL). As shown in Figure 5, MFC for NaTNT had a good antifungal efficacy against Penicillium and Mucor (27 and 31 µg/mL, respectively). Other examined isolates were likewise impacted by NaTNT, but at greater doses. 

**Figure 6:**
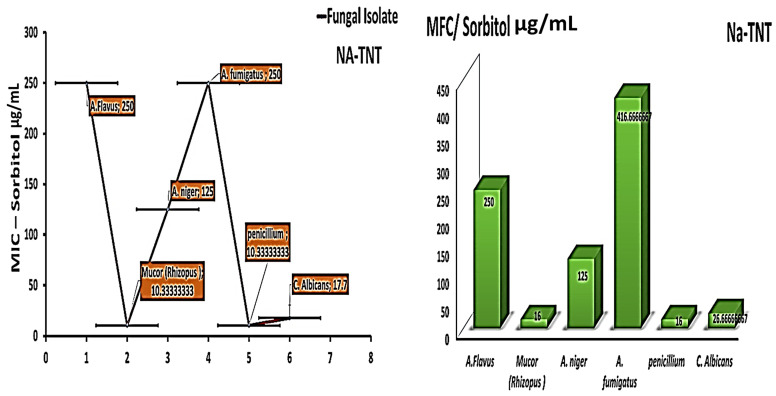
Presents the MIC and MFC values (µg/mL) of NaTNT on Sorbitol media against both different fungal isolates (Mean ± SE). The best obtained results for NA TNT were obtained on the Mucor and pencillieum species while fumigatus and flavus needed higher concentrations, MIC results with sorbitol decreased, indicating its action on the fungal cell wall.

To investigate the antifungal activity of the tested nanomaterial (mechanism of action of NaTNT as an antifungal agent), the experiment was conducted, repeated, and quantified using sorbitol. The MIC data were repeated on a different sorbitol medium to clarify the specific mechanism of action of the tested substances against various fungal strains, the lower values of effective MIC are an indication of the impact on the fungal cell wall. After evaluating the MIC in various mediums with more added sorbitol, the findings indicated that NaTNT was more active against Penicillium, Mucor, and Candida at concentrations of 10, 10, and 17 µg/mL, as opposed to the previously reported higher values in the current normal MIC (15.20 & 66 µg/mL, respectively). Figure 6. Sorbitol is an osmotic stabilizer used to stabilize the protoplasts of fungi. Specific fungal cell wall inhibitors have the property that their antifungal activities are nullified in sorbitol-containing media [47]. In the presence of fungal cell wall inhibitors, cells protected by sorbitol may proliferate, but growth would be impeded in the absence of sorbitol. This impact is recognized by the decrease in MIC values reported in media containing sorbitol compared to media without sorbitol (standard medium) [48]. Osmotic destabilizing agents and disruption of the cell wall result in the reorganization of the cell wall, allowing for the survival of fungal cells [49]. The studied substances seemed to operate on the cell wall, altering its structure, limiting its manufacture, and inducing cell death, as well as impeding spore germination, proliferation, and cellular respiration. 

In addition to lower effective fungicidal concentrations against Mucor and Penicillium, the investigated MFC findings utilizing various mediums with additional sorbitol concentrations revealed lower fungicidal concentrations against Mucor and Penicillium. As also shown in Figure 6, for Penicillium and Mucor, the MFC concentrations decreased to 16 and 16 µg/mL from 27 and 31 µg/mL for the other fungal strains. 

**Figure 7:**
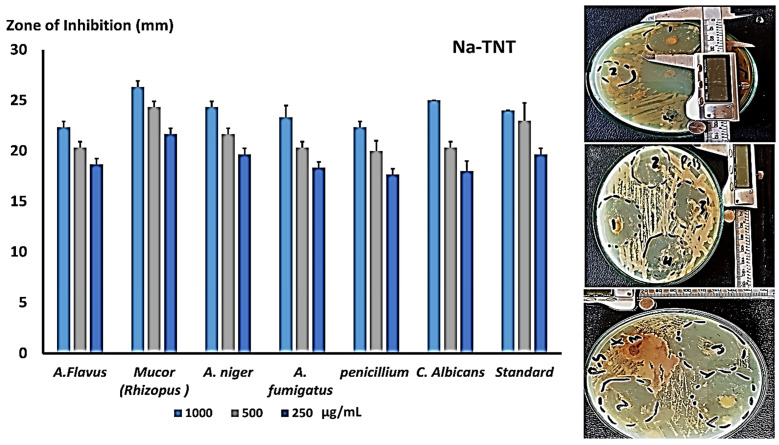
Illustrates the calculated mean of the inhibition zone (mm) at different concentrations of NaTNT versus diverse species of fungi, and the mean of the inhibition zone against standard antibiotics (cyclohexamide) (Mean ± SE). Representative clear zone of inhibition appeared in the agar plates. “1–4”: different testes concentration 1 = 1000; 2 = 500 & 3–4 = 250.

The disc diffusion method is regarded as one of the most precise techniques for measuring antifungal or antibacterial activity. On SDA plates, inhibition zones against several fungal strains were tested, and numerous concentrations are shown in Figure 7. They were evaluated at doses of 1000, 500, and 250 µg/mL; NaTNT demonstrated a good zone of inhibition against Mucor, Candida, and *A. niger* in comparison to the conventional antifungal for rapidly growing fungus (cyclohexamide). As shown in Figure 7, NaTNT showed promising antifungal effectiveness against the other fungal strains. 

**Figure 8:**
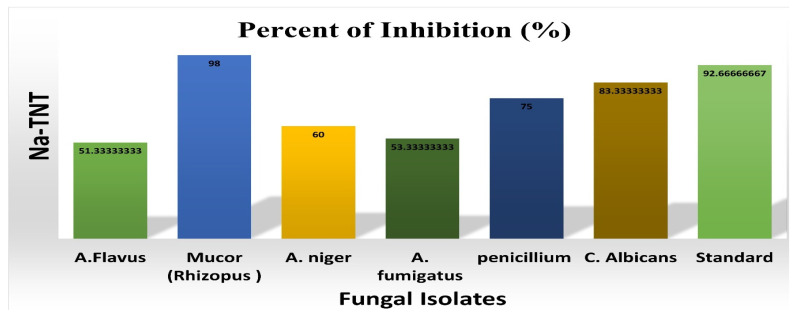
Percent of inhibition (%) of NaTNT in SDA media against multiple fungal strains. The best inhibitory percentage was against the Mucor type.

Concerning antifungal activity (inhibition percentage), on the basis of the measurement of antifungal activity following the addition of materials to media, the percentage of fungal inhibition was calculated and shown in Figure 8. Mucor was inhibited at a rate of 98% by NaTNT, whereas candida was inhibited at a rate of 83%. The antifungal action of NaTNT is a result of its unique features, including its tiny size, wide surface area, and uniform dispersion.

**Figure 9:**
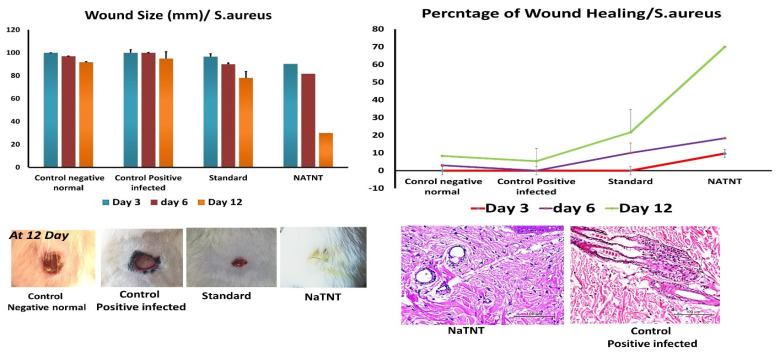
Size of the infected wound with *S. aureus* (10^8^ CFU/mL), percentages of wound healing, and histopathological investigations (Mean ± SE). Percentages of wound healing were the best obtained for Na TNT at day 12 of the experiment when compared to the standard and control positive groups.

After investigating the distinctive structure of NaTNT, we assessed it’s in vitro and in vivo antibacterial activities. The in vitro antibacterial activity of NaTNT was evaluated using the conventional colony counting technique on two different microorganism strains, one bacterial (*S. aureus*) and the other a severe fungus isolate (*M. Rhizopus*). In this study, the (in vitro) antimicrobial activity of NaTNT displayed outstanding inhibitory activity against both *S. aureus* and *Mucor rhizopus* (in vitro study). Using rats with *S. aureus* and *Mucor rhizopus*-infected skin lesions as a model, we next examined the in vivo antibacterial effectiveness of NaTNT. Photographs of the wounds of rats in the control group and the NaTNT group at 3, 6, and 12 days were shown in Figure 9. Compared to the control groups, the NaTNT group demonstrated a significant decrease in wound area after 3 days. Comparably; after 12 days, wound area reduced to 5% in the NaTNT group, suggesting the high antibacterial action of NaTNT. In addition, the number of CFU in the NaTNT group after 12 days was significantly lower than in the control group with 0% inhibition percentages (Figure 9), further proving their antibacterial activity against multidrug-resistant bacteria in vivo. These findings indicated that NaTNT, a new antimicrobial agent, had excellent antibacterial and antifungal properties.

**Figure 10:**
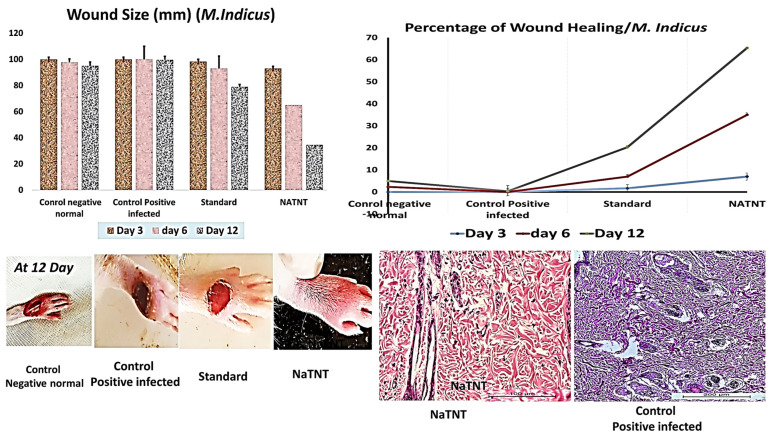
Size of the infected wound with *M. Rhizopus* (10^8^ CFU/mL), percentages of wound healing, and histopathological investigations. (Mean ± SE). Higher healing percentages for the infected wound was achieved by Na TNT at the 12th day of treatment when compared to other groups.

**Figure 11:**
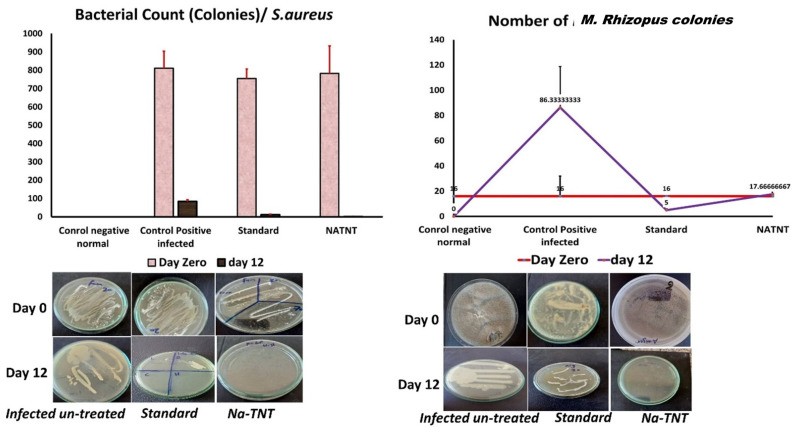
Bacterial and fungal colonies from the infected wound at Day Zero and on the last day of treatment (Day 12). (Mean ± SE). No colony growth appeared on the 12th day in the Na TNT groups. The highest bacterial colonies were obtained in the control positive infected non-treated group.

The wound pictures of rats in each group at 3, 6, and 12 days for NaTNT antifungal activity were shown in Figure 10. Compared to the normal control and infected untreated groups, the NaTNT group showed a significant decrease in wound area after 3 days. After 6 days, the wound area in the NaTNT group reduced below 20% (Figure 10), indicating more rapid healing. In addition, wound tissues from each group were collected at 0 and 12 days. At 12 days, the number of colony-forming units (CFU) in the NaTNT group was much lower than in the control and standard groups (Figure 11), revealing the remarkable antibacterial activity of NaTNT against in vivo multidrug-resistant bacteria. Additionally, we evaluated the antibacterial therapeutic effects of NaTNT by histological examination. After 12 days of therapy, rats in each group had their wounds extracted for H&E staining. On H&E staining, there are fewer infected tissues in the NaTNT group than in the control infected group (Figure 9 and Figure 10). Moreover, throughout the wound’s healing phase, these findings indicated that NaTNT exhibited remarkable antibacterial activity in vivo, indicating its considerable potential for treating illnesses caused by bacteria that are resistant to many drugs. NaTNT resulted in full wound healing with normal epithelial formation and vasculature, as determined by histopathological examinations of skin samples from the various treatments. In addition, the same healing activity was seen in a conventional group, albeit at a lesser efficiency than in the two experimental groups. Other untreated groups of rats had inadequate wound healing, including congestion and epithelial rupture (Figure 9 and Figure 10).

During daily life, microorganisms such as bacteria, fungi, and viruses often infect humans. The use of antimicrobial drugs is essential for imparting sterility (e.g., hospital trays) and avoiding infection (e.g., wound dressing) along one path to bone infection. Antibacterial compounds, including a variety of natural and inorganic substrates, have been extensively researched [19]. TiO_2_ has gained more attention than other antimicrobial chemicals due to its superior stability, environmental friendliness, safety, and extensive antibiosis [20]. The use of tiny TiO_2_ particles in antibacterial compositions has been the topic of much investigation. Nonetheless, the powder TiO_2_ catalyst has the problem of slurry separation post-application following photoreaction [20]. Consequently, surface-immobilized TiO_2_ with a high specific surface area is more advantageous for antibacterial applications [21].

Wound healing and bone affections continue to be demanding clinical issues for which effective wound management and care are required. Moreover, successful wound and tissue regeneration remains a significant healthcare and biological problem in the twenty-first century. In addition to being a burden on the healthcare system’s resources, infected or chronic wounds often result in death due to the inability to fulfil the targeted function and the rise in pain intensity [50]. Therefore, the discovery of procedures or medications that may aid in expediting the wound healing process and decreasing the time required for full wound recovery would reveal a treatment of major value. In addition to histopathological results, the percentage of healing activity and the size of wound closure over the course of 12 days were employed as a marker for wound healing activity. NaTNT demonstrated a quick and complete wound healing process in a shorter period of time than the standard alone and the untreated group after topical application. The effective healing properties of NaTNT may be linked to the nanomaterial’s high wound-penetrating ability, which is a result of its enhanced surface area. Infections with bacteria are the primary cause of unhealed wounds [51]. Thus, the hunt for new materials that are effective against Gram-positive and Gram-negative bacteria is crucial. Staphylococcus is one of the most prevalent bacterial pathogens responsible for the vast majority of wound infections and is one of the leading causes of hospital-acquired infections. Bacteremia, sepsis, and/or toxic shock syndrome may result from the ineffectiveness of antimicrobial drugs or their low invading capability [52]. Histopathological studies revealed that the NaTNT exhibited rapid contraction, which is crucial for the quick healing of wounds, particularly in animals with loose skin (mice, rats). Re-epithelialization was a common step in the wound healing process and contact epithelial surface for all species. To the best of our knowledge, no prior trials on the effectiveness of NaTNT for wound infections have been published [53].

The benefits of topical treatment include the capacity to give a high local concentration with minimal dosages of the drug, especially in patients with limb ischemia, so as to avoid the first-pass action in the gastrointestinal tract and minimize the risk of systemic adverse effects. Very high local concentrations are achieved using topical preparations [54]. No clinical evidence supports the use of topical treatments for the prevention of infection recurrence in bone or wound or bone pathologies. All open wounds are colonized by bacteria, which impact the healing process in general. However, if the colonization progresses into a local infection, which then becomes systemic, the outcome might be fatal. Therefore, wound care includes not only cleaning, debridement, and therapy of the underlying etiology, but also steps to reduce the likelihood of colonized wounds becoming locally or even systemically infected in order to avoid osteomyelitis or arthritis [55]. Local antibiotic therapies have been shown to promote healing, however, treatment of the underlying etiology remains essential [56]. The number of bacteria present on a wound’s surface is a local factor that might impede healing.

Despite the fact that the actual mechanism or interaction between metal inorganic framework structures and various infections has never been described, several demonstrations might be proposed. Consistent with earlier research, the inhibitory antibacterial activity of NaTNT may be attributed to the generation of reactive oxygen species. *M. Rhisopus* and *S. aureus* have their adhesion inhibited in nanotube films due to the high wettability and roughness of the nanoscale of the films. The antibacterial activity of NaTNT may have been due to the spontaneous release of free radicals such as ROS, inducing oxidative stress-mediated cell damage. Consider the possibility that cell membrane damage was generated by the electrochemical manner of interaction between the Na+ ions and the phosphate group in the lipid layers, hence weakening cell membrane integrity and generating membrane leakage. Similar to the suggested process, Gram-positive bacteria with numerous pores (Porins) may have let NaTNT enter into the cell, leading to membrane disruption, cell content release, and eventually cell death [57]. 

Similar to that of bacteria, the antifungal activity of NaTNT may be the result of the electrostatic interaction between the phosphate group in the cell membrane and Na+, the penetration of NaTNT into the cell, and the subsequent binding of Na+ with the thiol group of proteins, which results in their denaturation. In addition, NaTNT may have triggered cell death through ROS-mediated oxidative stress. These assumptions need close examination. On the other hand, NaTNT may play a substantial role, in particular, its antibacterial efficacy has been proven. In addition, it was shown that NaTNT caused permanent alterations in membrane characteristics (charge, intra and extracellular permeability, and physicochemical properties). Changes in cell surface hydrophobicity, charge, increased PI uptake, and K+ leakage with local rupture or hole formation in the cell membranes of Gram-negative and -positive bacteria were established as the mechanism for the antibacterial action of NaTNT [58].

These findings are consistent with earlier research about the precise mechanism of action for the antibacterial activity of NaTNT. The production of reactive oxygen species (ROS) such as the hydroxyl radical explains the antibacterial action of nanomaterials based on titanium. The antibacterial action of -OH radicals is linked to their capacity to destroy the cell wall. Thus, it stimulates its perforation, destruction of the cytoplasmic membrane, and cell lysis, resulting in the organism’s total mineralization. Additionally, H_2_O_2_ may permeate the cell membrane, react with biological macromolecules, and serve as a precursor to -OH. Therefore, the production of ROS may account for the enhanced antibacterial action of the tested nanomaterials. Kuhn et al. [59] demonstrated that the superior antibacterial activity was due to the direct action of the ROS generated by the photocatalytic process, which caused peroxidation of the lipid components of the cell wall, including lipopolysaccharides, phospholipids, and lipoproteins of the outer membrane, as well as plasma membrane phospholipids [60].

Hydrophobicity plays a crucial role in the attachment of several bacterial species to their substrates, which is another factor for the antibacterial action of NaTNT [11,47]. According to studies, there is significant interaction between bacteria and hydrophobic surfaces, resulting in high cell attachment rates [61]. In a hospital setting, bacterial adherence to biotic and abiotic surfaces may be a cause of dangerous infections [61]. Therefore, it is desirable to produce super hydrophilic surfaces that reduce the contact between bacteria and substrates. In a physiological environment, super hydrophilic surfaces create a thin layer of water that may prevent the development of bacterial biofilms by inhibiting bacterial adhesion [62]. Ji et al. [18] found that increasing the hydrophilicity of titanium surfaces decreased *E. coli* adherence. Additionally, surface roughness might affect bacterial adhesion. According to studies, nanoscale roughness has anti-adhesive properties. This was found in research on both Gram-negative and Gram-positive bacteria. Thus, the roughness of the nanotube films may have also contributed to their ability to impede bacterial attachment [63].

In this research, sodium titanate nanotubes (NaTNT) had the highest antibacterial activity; few studies have indicated antimicrobial activity for titanate nanostructures. Titanate nanotubes show substantial antibacterial activity against Gram-positive bacteria and also Gram-negative bacteria, but at a lesser level based on the observed zone of inhibition as compared to Gram-positive bacteria, as reported by Kundu et al. in prior research [64]. Titanate nanotubes have a considerable antibacterial impact against *Bacillus subtilis*, which may be due to their interaction with the microorganism’s cell wall composition. In contrast to the findings of Kundu et al., this investigation demonstrates that the activity of titanate nanostructures against Gram-positive bacteria is not necessarily superior to that against Gram-negative bacteria [64]. The interaction between nanostructures and the bacterial cell wall is mostly driven by the zeta potential. Regardless of the surface area and crystallite size, the surface charge will influence the electrostatic attraction or repulsion between the nanostructures’ surface and the bacterial cell wall. This will determine the kind of interaction (bacteria/fungi—nanoparticles) and, therefore, the MIC for each strain of bacteria. Finally, NaTNT is the most effective agent for treating both Gram-positive and Gram-negative bacteria.

In conclusion, NaTNT structures enhance several research applications; the current work focused on one of these structures, namely NaTNT in terms of synthesis, characterization, and application. The study attempted to evaluate the antibacterial efficacy of NaTNT against types of serious disease-causing microorganisms such as Mucor and staph that damage bone and joints by entering infected wounds. In light of this, the research implies that NaTNT has the potential for wound protection against both bacterial and fungal infections.

Gram-positive and mucormycosis infections are responsible for the majority of bone and joint infections, particularly in immunocompromised individuals. Antibiotics penetrate effectively into the synovial fluid of infected joints, and after drainage, septic arthritis may be treated with two to three weeks of intravenous and oral medication. Nanomaterial penetration into bone is more varied and reliant on a number of variables. In addition to the removal of all contaminated tissue, osteomyelitis treatment needs weeks to months of antibiotic treatment. Traditionally, the whole course of parenteral antibiotics has been recommended to attain adequate bone concentrations. The ultimate choice of antibiotic, usage of oral treatment, and course length are determined by microbiological, surgical, and patient considerations, and should be reviewed with the physician and medical microbiology for each individual patient. The prevalence of more resistant infecting organisms is worrisome, both in terms of patient care and broader implications for cross-infection. Although the first studies are promising, the effectiveness of the ‘new’ antibiotics in treating orthopaedic infections has yet to be shown.

## 4. Future Study

In the future, the most effective and commonly used coating material for immune-compromised patients with COVID-19 or other immunosuppressive diseases, as well as diabetic patients, will be nano-synthesized NaTNT. This material has been proven to have antifungal and antibacterial properties against life-threatening mucormycoses and other bacterial or fungal infections that can cause bone damage.

## Figures and Tables

**Table 1:** Textural parameters of Na-TNT.

S_BET_ (m^2^·g^−1^)	Average Pore Size (nm)	Total Pore Volume (cm^3^/g)
154.85	5.791	0.21732

## Data Availability

All data are provided in the manuscript.
